# The Cholesterol-Lowering Effect of Capsella Bursa-Pastoris Is Mediated via SREBP2 and HNF-1α-Regulated PCSK9 Inhibition in Obese Mice and HepG2 Cells

**DOI:** 10.3390/foods10020408

**Published:** 2021-02-12

**Authors:** Jin-Taek Hwang, Eunji Choi, Hyo-Kyoung Choi, Jae-Ho Park, Min-Yu Chung

**Affiliations:** 1 Korea Food Research Institute, Wanju-gun, Jeollabuk-do 55365, Korea; jthwang@kfri.re.kr (J.-T.H.); chkyoung@kfri.re.kr (H.-K.C.); jaehopark@kfri.re.kr (J.-H.P.); 2 Department of Food Biotechnology, Korea University of Science & Technology, Daejeon 34113, Korea; 3 Department of Food and Nutrition, Sookmyung Women’s University, Seoul 04524, Korea; choieunji@sookmyung.ac.kr

**Keywords:** capsella bursa-pastoris, PCSK9, LDL receptor, SREBP2, statins, icaritin

## Abstract

The objective of the present study was to investigate the mechanism by which capsella bursa-pastoris ethanol extract (CBE), containing 17.5 milligrams of icaritin per kilogram of the extract, and icaritin, mediate hypocholesterolemic activity via the low-density lipoprotein receptor (LDLR) and pro-protein convertase subtilisin/kexin type 9 (PCSK9) in obese mice and HepG2 cells. CBE significantly attenuated serum total and LDL cholesterol levels in obese mice, which was associated with significantly decreased PCSK9 gene expression. HepG2 cells were cultured using delipidated serum (DLPS), and CBE significantly reduced PCSK9 and maintained the LDLR level. CBE co-treatment with rosuvastatin attenuated statin-mediated PCSK9 expression, and further increased LDLR. The icaritin contained in CBE decreased intracellular PCSK9 and LDLR levels by suppressing transcription factors SREBP2 and HNF-1α. Icaritin also significantly suppressed the extracellular PCSK9 level, which likely contributed to post-translational stabilization of LDLR in the HepG2 cells. PCSK9 inhibition by CBE is actively attributed to icaritin, and the use of CBE and icaritin could be an alternative therapeutic approach in the treatment of hypercholesterolemia.

## 1. Introduction

Cardiovascular diseases are a major cause of death worldwide [1]. Increased serum low-density lipoprotein (LDL) cholesterol is one of the major risk factors for these diseases [2]. Statins are the most widely prescribed drugs for the treatment of hypercholesterolemia. As a 3-hydroxy-3-methylglutaryl-coenzyme A (HMG-CoA) reductase inhibitor, statins increase the nuclear translocation of sterol regulatory element binding protein 2 (SREBP2), which is a transcription factor of the LDL receptor (LDLR) and proprotein convertase subtilisin/kexin type 9 (PCSK9). Therefore, statins enhance the expression of both PCSK9 and LDLR [3,4].

PCSK9 interferes with the binding between LDL cholesterol and LDLR at the cell surface, and subsequently promotes the lysosomal degradation of LDLR [5]. Owing to this negative feedback response of PCSK9 on the LDL cholesterol-lowering effect of LDLR, PCSK9 inhibition has been suggested as an alternative strategy for the treatment of hypercholesterolemia and related-chronic diseases since its discovery in 2003 [6,7]. Several clinical trials have supported this idea. For instance, monoclonal antibodies against PCSK9 (Evolocumab and Alirocumab) [8,9,10] have been shown to decrease LDL cholesterol levels in Phase II and III clinical trials [11,12]. Furthermore, two large-scale randomized clinical trials, namely FOURIER and ODYSSEY, have been performed using fully human anti-PCSK9 monoclonal antibodies to evaluate the PCSK9-mediated pathway [13,14]. Both the FOURIER and ODYSSEY trial outcomes demonstrated that cardiovascular events are improved by further lowering of LDL cholesterol through co-treatment with statin therapy [13,14]. Therefore, PCSK9 inhibition can be suggested as a new target for the treatment of cardiovascular patients who do not achieve optimal LDL cholesterol levels with statin treatment alone.

However, a number of questions regarding the use of PCSK9 inhibitors as a new class of drugs remain unanswered. The cost of PCSK9 inhibitors is high compared to that of other lipid-lowering drugs. Indeed, the annual cost of PCSK9 antibodies (Evolocumab and Alirocumab) treatment in the USA is over 14,000 dollars [15]. Long-term safety and mortality reduction of PCSK inhibitors still remain subjects of investigation. Additionally, PCSK9 drugs need to be parenterally administered. Hence, foods and/or their active compounds that may target PCSK9 have no safety issues, and their oral availability may be a desirable alternative strategy.

*Capsella bursa-pastoris*, also known as Shepherd’s purse, has been reported to have a number of health benefits, including antioxidant activity via the reduction of reactive oxygen species production, and anti-inflammatory activity via the inhibition of the production of prostaglandin E2, tumor necrosis factor-α, and interleukin-6 [16]. Our previous study also demonstrated that an ethanol extract of *Capsella bursa-pastoris* (CBE) protects against hepatic steatosis by inhibiting histone acetyltransferase (HAT) activity in HepG2 cells, and in a diet-induced obese mouse model [17]. Additionally, we previously found that 2% (*wt/wt*) CBE supplementation in a high-fat diet in an obese mouse model significantly attenuated the total cholesterol levels of the experimental group, compared to those of the control group [17].

To the best of our knowledge, there are no studies demonstrating *Capsella bursa-pastoris*’s effects on the regulation of PCSK9 in hypercholesterolemia. We performed a screening study using up to 40 different food materials, including food extracts and single compounds, and observed that CBE exhibited greater PCSK9 inhibition. Therefore, the aim of the present study was to investigate the cholesterol-lowering effect of lower levels of CBE (1%, *wt/wt*), and the mechanism by which CBE mediates PCSK9 and LDLR in a diet-induced obese mouse model and HepG2 cells. We also investigated the synergistic effect of CBE and statins in HepG2 cells, the inhibitory effect of PCSK9 by CBE’s active compounds, and the underlying mechanisms of these effects.

## 2. Materials and Methods

### 2.1. Reagents

Corning^®^ High-Glucose Dulbecco’s Modified Eagle Medium (DMEM) was purchased from Mediatech, Inc. (Manassas, VA, USA). Isopropanol and chloroform were obtained from EMD Millipore (Billerica, MA, USA). Cycloheximide, bafilomycin A1, thiazolyl blue tetrazolium bromide (MTT), acacetin (purity ≥ 97%), sinensetin (purity ≥ 98%), and icaritin (purity ≥ 98%) were purchased from Sigma-Aldrich (St. Louis, MO, USA). Dimethyl sulfoxide (DMSO) was purchased from Duchefa Biochemie (Haarlem, Netherlands). Fetal bovine serum (FBS), distilled phosphate buffered saline (DPBS), and antibiotic and anti-mycotic agents were purchased from WELGENE Inc. (Gyeongsan, Republic of Korea). CircuLex™ polyclonal anti-PCSK9 antibody was purchased from Medical & Biological Laboratories Co., Ltd. (Nagoya, Japan). Polyclonal anti-LDLR was purchased from BioVision Inc. (Milpitas, CA, USA). Monoclonal anti-HNF1α was purchased from Cell Signaling Technology (Cell Signaling Technology, Inc., Danvers, MA, USA), and polyclonal anti-SREBP2 was supplied by Cayman Chemical (Ann Arbor, MI, USA). Polyclonal anti-β-actin was purchased from Bethyl Laboratories (Montgomery, TX, USA). Peroxidase-conjugated purified goat anti-rabbit IgG was purchased from Enzo Life Sciences, Inc. (Farmingdale, NY, USA). High performance liquid chromatography (HPLC) grade water and methanol were purchased from Avantor Performance Materials (Center Valley, PA, USA).

### 2.2. Animals and Diet

The protocol for the care and use of animals was approved by the Institutional Animal Care and Use Committee at Wonkwang University (#WKU16-21). Male C57/BL6 mice (7 weeks old) were housed in a temperature- and humidity-controlled room, with a 12 h light/12 h dark cycle. After 1 week of acclimation, the mice were fed with either a western-type diet (WD) or a low-fat diet (LD, *n* = 8). The mice fed with the WD were divided into two dietary groups. One group was fed with WD only (*n* = 8), and the other group was fed with WD supplemented with 1% CBE (*wt/wt*; WD+CBE, *n* = 9). Isocaloric LD (D14042701) and WD (D12079B) were purchased from Research Diets (Research Diets, Inc., New Brunswick, NJ, USA). For the LD, 10% of energy comes from fat, 73% from carbohydrates, and 17% from protein. For the WD, 40% of energy comes from fat, 43% from carbohydrates, and 20% from protein. The diet composition has been described in our previous study [18]. The CBE was prepared using 70% ethanol via the processes of agitation, centrifugation, and freeze-drying. Food and water were provided *ad libitum*. Food intake and body weight changes were monitored weekly. After 12 weeks of feeding, the mice were starved overnight (10–12 h), and blood was collected from the retro-orbital sinus. Livers were harvested following cervical dislocation, rinsed with saline solution, snap frozen, and stored at −80 °C until analysis. The masses of body and organs, and food intake have been partially reported in our previous study [18].

### 2.3. Total and Low-Density Lipoprotein Cholesterol

Serum was obtained by centrifugation (430× *g*, 10 min, 4 °C). Serum total and LDL cholesterol levels were measured using enzymatic colorimetric assay kits (Asanpharm, Hwasung, Korea) in a Modular Analytics machine (model PE, Roche Diagnostics, Mannheim, Germany).

### 2.4. HepG2 Cell Culture and Treatment

HepG2 cells (HB-8065; ATCC, Manassas, VA, USA) were cultured in high-glucose DMEM supplemented with 10% FBS (Welgene Inc.) and 1% antibiotic and anti-mycotic solution (Welgene Inc.). Delipidated (lipid deprivation) serum (DLPS) was prepared following a previous method [19]. In brief, butanol and isopropyl ether were added to FBS (WELGENE Inc., Gyeongsan, Republic of Korea). After stirring and cooling, the organic top layer was removed by aspiration. Mixing and aspiration were repeated. Following spinning, we transferred the lower layer to a clean flask and kept it under nitrogen gas for 2 h, further lyophilizing it until dry (more than 24 h). The obtained powder was rehydrated in distilled water to about 40 mg/mL protein, which was dialyzed. Protein amount, cholesterol, triacylglycerol, and fatty acid levels were determined to confirm the lipids were removed. After reaching 70–80% confluence, the cells were seeded in well plates (day 0), and the medium was changed to either DMEM supplemented with FBS or DMEM supplemented with DLPS (day 1). After 24 h incubation, the medium was changed to media supplemented with either FBS or DLPS, and simultaneously treated with either samples (CBE or chemical compounds) or DMSO (Duchefa Biochemie) (day 2). After an additional hour of incubation (day 3), the cells were either washed with cold DPBS, or collected for subsequent experiments.

### 2.5. Cell Viability

Cell viability was measured using a thiazolyl blue tetrazolium bromide (MTT; Sigma-Aldrich) solution. After the cells were cultured, seeded, and treated as described above, the cells were washed with cold DPBS, and DMEM with no FBS supplementation was added into each well. Five mg/mL of MTT solution (10 µL/well) was added, and the cells were incubated for an additional 2–3 h. A mixture of isopropanol (EMD Millipore) and DMSO (1:1, 50 µL) was added into the wells, followed by incubation at room temperature for 5 min with gentle shaking. The absorbance was then read at 570 nm (Molecular Devices, Sunnyvale, CA, USA).

### 2.6. ELISA

To measure the protein concentrations of PCSK9 and LDLR, HepG2 cells were seeded and treated as described above and centrifuged (100× *g*, 3 min); pellets were harvested following washing with cold DPBS. LDLR (STA386) and PCSK9 (STA385) concentrations in the HepG2 cell lysates were measured using commercially available ELISA kits, in accordance with the manufacturer’s instructions (Cell Biolabs, Inc., San Diego, CA, USA) [20]. Intracellular and extracellular PCSK9 and LDLR were normalized by total protein (Bio-Rad Laboratories, Hercules, CA, USA) level in whole cell lysates.

### 2.7. Western Blot

To analyze the protein expression levels of LDLR, PCSK9, SREBP2, and HNF-1α, HepG2 cells were seeded and treated as described above, and the cells were washed with cold DPBS, harvested, and centrifuged (1200 rpm, 3 min). The collected pellet was lysed with the RIPA buffer (Elpis Biotech, Inc.) containing protease and phosphatase inhibitors (Roche, Basel, Switzerland). The lysates containing equal amounts of protein, were then loaded on Bis-Tris gels for electrophoresis (10%), before being transferred to nitrocellulose membranes. The blots were blocked in 5% skim milk solution for 1 h and probed with specific antibodies (PCSK9 (MBL Life Science, Woburn, MA, USA), LDLR (Biovision Inc., Milpitas, CA, USA), HNF-1α (Cell Signaling Technology, Inc., Danvers, MA, USA), SREBP2 (Cayman Chemical, Ann Arbor, MI, USA), and β-actin (Bethyl Laboratories Inc., Montgomery, TX, USA)]. After washing three times with tris-buffered saline (TBS) with Tween 20, the blots were incubated with peroxidase-conjugated purified goat anti-rabbit IgG (Enzo Life Sciences, Inc., Farmingdale, NY, USA) for 1 h. After an additional wash with TBS with Tween 20, the proteins were detected using chemiluminescence reagent (Pierce Biotechnology, Rockford, IL, USA). The chemiluminescent signal was analyzed using the Quantity One software (Bio-Rad Laboratories, Hercules, CA, USA).

### 2.8. Quantitative Real-Time PCR

Following the treatment of the cells as described above, the collected cell pellets were lysed with Isogen (Takara Bio, Inc., Kusatsu, Japan); the mouse liver samples were also lysed with Isogen. After the addition of an equal volume of chloroform (EMD Millipore), the upper layer of the lysing solution was collected. After centrifugation (1600× *g*, 10 min, 4 °C), the upper layer was again collected and mixed with an equal volume of isopropanol (EMD Millipore) to precipitate RNA. Reverse transcription was performed using a cDNA reverse transcription kit (Takara Bio). Quantitative real-time PCR was performed in a reaction mixture containing cDNA, primers (10 pmol), and SYBR Green (Roche, Basel, Switzerland). The GenBank database was used to design the primers as per methods described previously [20]. PCR amplification was performed (Bio-Rad Laboratories, Hercules, CA, USA) under the following initial denaturation, denaturation, annealing, and elongation conditions: 95 °C for 10 min, 95 °C for 30 s, 56 °C for 45 s, and 72 °C for 45 s, respectively (data collection). The ΔΔCt method was used to analyze the data [21], and all mRNA expression levels were normalized to that of GAPDH.

### 2.9. HPLC Analysis

The polyphenolic compounds in CBE, namely acacetin, sinensetin, and icaritin, were identified and quantified using HPLC according to the method described by Vandercook and Tisserat (1989), with the following modifications. The CBE sample (~400 mg) was added to a 50 mL conical tube containing 40 mL of DMSO and mixed using a vortex mixer for 2 min at room temperature. The sample was centrifuged (3000 rpm, 15 min) and filtered through a PVDF syringe filter (0.45 μm, Woongki Science, Seoul, Korea). A Waters HPLC system (Waters, Milford, MA, USA), equipped with an e2695 Separations Module and a 2998 photodiode array detector, was used for the analysis. The separation was performed on a Capcell pak C18 MG2 column (250 mm × 4.6 mm i.d., Shiseido, Tokyo, Japan) maintained at 40 °C. Optimal separation was achieved by gradient elution using mobile phase (A) 0.01 M H_3_PO_4_ and (B) methanol at a flow of 0.6 mL/min. The gradient system consisted of an initial 2 min of 80% solvent A and 20% solvent B followed by a linear gradient to 100% solvent B in 60 min, and a linear gradient to 80% solvent A and 20% solvent B in 80 min. The detector measured spectra from 200 to 380 nm and monitored the eluent at 265 nm, which is the wavelength of maximum absorption for the three polyphenolic compounds. Each compound was identified by comparing its retention time (sinensetin, ~39.1 min; acacetin, ~42.5 min; and icaritin, ~53.5 min) with that of the reference standard, and its concentration was calculated as milligrams per kilogram of CBE. The standard solutions (concentrations of 0.025, 0.05, 0.1, 0.25, 0.5, 1, and 2 mg/L) for calibration were prepared from a stock solution of 200 mg/L by dilution. Each measurement was performed in triplicate.

### 2.10. Statistical Analysis

Data are expressed as the mean ± SD, and the results were analyzed using Student’s *t*-test to compare mean differences between groups. Data were analyzed using the GraphPad Prism software (La Jolla, CA, USA), and *p*-values of <0.05 were considered statistically significant.

## 3. Results

### 3.1. Hypocholesterolemic Activity of CBE Was Attributed to Decreased PCSK9 Gene Expression in WD-Fed Obese Mice

Mice were fed with the LD, WD, or WD diet supplemented with CBE at 1% (*wt/wt*) for 12 weeks. In concurrence with a previous study [17], CBE exhibited a cholesterol-lowering activity. CBE at 1% significantly attenuated (*p* < 0.01) total and LDL cholesterol in obese mice fed with WD compared to obese controls (Table 1).

To investigate whether the hypocholesterolemic activity of CBE can be attributed to defective LDLR in the liver, *LDLR, SREBP2, HMGCR,* and *PCSK9* gene expression levels were measured using qRT-PCR. Interestingly, *LDLR, SREBP2,* and *HMGCR* gene expression levels were affected by neither diet nor CBE in obese mice, while the *PCSK9* gene alone was significantly (*p* < 0.05) reduced by CBE supplementation in obese mice (Table 2). Therefore, the CBE-mediated cholesterol-lowering effect observed in obese mice is associated with PCSK9 inhibition.

### 3.2. CBE Significantly and Markedly Suppressed the Levels of Intracellular PCSK9, but Not LDLR, in HepG2 Cells under Lipid Depletion Conditions

**Figure 1:**
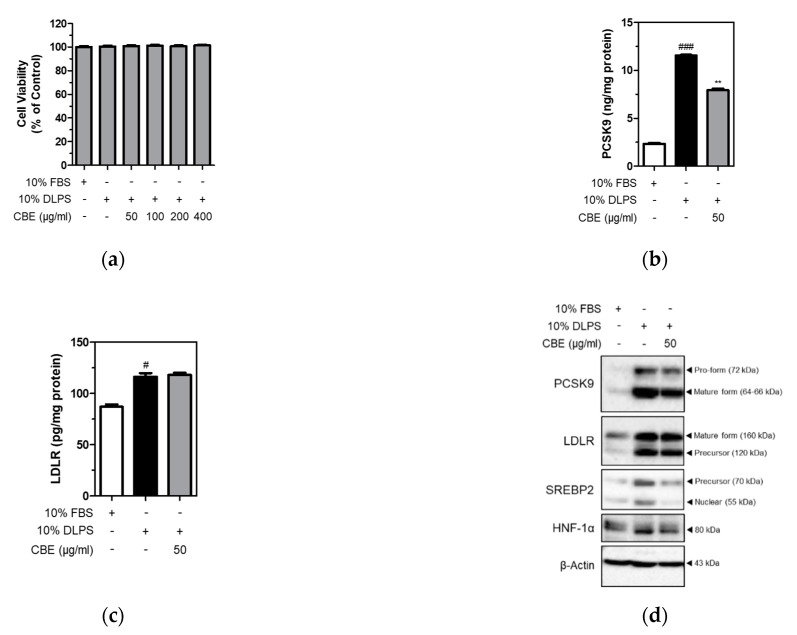
Viability of HepG2 cells in the presence of CBE (50, 100, 200, 400 μg/mL) under lipid depletion conditions (**a**). Intracellular PCSK9 (**b**) and LDLR (**c**) levels were measured using ELISA. PCSK9 and LDLR, and the transcription factor SREBP2 and HNF-1α protein expression (**d**) were measured using Western blot analysis in HepG2 cells treated with FBS, DLPS, or DLPS+CBE (50 μg/mL). significant difference between cells treated with FBS and DLPS (Student’s *t*-test, # *p* < 0.05; ### *p* < 0.001). significant difference between cells treated with DLPS and CBE (Student’s *t*-test, ** *p* < 0.01).

To further examine PCSK9 inhibition by CBE, HepG2 cells were cultured under lipid depletion conditions in the presence or absence of CBE. MTT assay was performed to measure cell viability. Cell viability was not affected by either DLPS or CBE of up to 400 µg/mL in HepG2 cells (Figure 1a). Intracellular PCSK9 and LDLR levels were measured in HepG2 cells using ELISA. DLPS significantly (*p <* 0.001) increased intracellular PCSK9 level, and CBE at 50 µg/mL significantly (*p <* 0.01) attenuated intracellular PCSK9 level in HepG2 cells (Figure 1b). LDLR levels, which were increased by DLPS treatment, were unaffected by CBE treatment (Figure 1c).

Consistently, results from Western blot analysis showed that DLPS increased both PCSK9 and LDLR protein expression (Figure 1d). CBE treatment also markedly reduced DLPS-mediated increases in PCSK9 protein expression, whereas LDLR expression was not affected by CBE in HepG2 cells. Protein expression of SREBP2 and HNF-1α, an independent transcription factor of PCSK9 was also measured. DLPS-mediated increases in SREBP2 and HNF-1α expression were markedly attenuated by CBE at 50 µg/mL treatment (Figure 1d). Taken together, CBE reduced intracellular PCSK9 expression, which was otherwise increased by DLPS treatment. This contributed to maintaining the intracellular LDLR level, likely by inhibiting LDLR lysosomal degradation in HepG2 cells. In addition, PCSK9 inhibition by CBE is likely mediated by decreased SREBP2 and HNF-1α in HepG2 cells.

### 3.3. CBE Synergistically Enhanced LDLR by Inhibiting PCSK9 Protein Expression in HepG2 Cells with Statin Treatment

**Figure 2:**
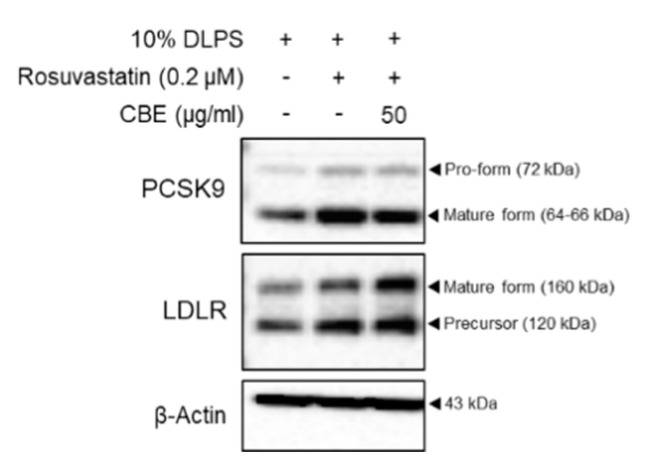
PCSK9 and LDLR protein expression in HepG2 cells treated with DLPS and rosuvastatin (0.2 µM) and mevalonate (50 µM) in the presence or absence of CBE (50 µg/mL), as measured by Western blot.

To examine whether CBE synergistically regulates PCSK9 and LDLR, rosuvastatin was added to DLPS in the presence or absence of CBE in HepG2 cells. Expectedly, rosuvastatin increased both PCSK9 and LDLR levels in DLPS-treated HepG2 cells (Figure 2). Treatment with 50 µg/mL CBE reduced the statin-mediated increases in PCSK9 protein expression compared to statin treatment alone in HepG2 cells treated with DLPS (Figure 2). CBE and statin co-treatment also further increased LDLR levels compared to those treated with rosuvastatin alone in DLPS-treated HepG2 cells (Figure 2). While treatment with CBE alone maintained a DLPS-mediated increase in the intracellular LDLR levels (Figure 1d), treatment with CBE in the presence of rosuvastatin further increased LDLR expression in HepG2 cells under lipid depletion conditions (Figure 2). Therefore, CBE and statin co-treatment has a synergistic effect, and CBE can be a useful supplement to statin with the help of a complementary action exerted via the inhibition of PCSK9.

### 3.4. Icaritin Is the Most Active Compound in CBE in the Regulation of PCSK9 and LDLR in HepG2 Cells

**Figure 3:**
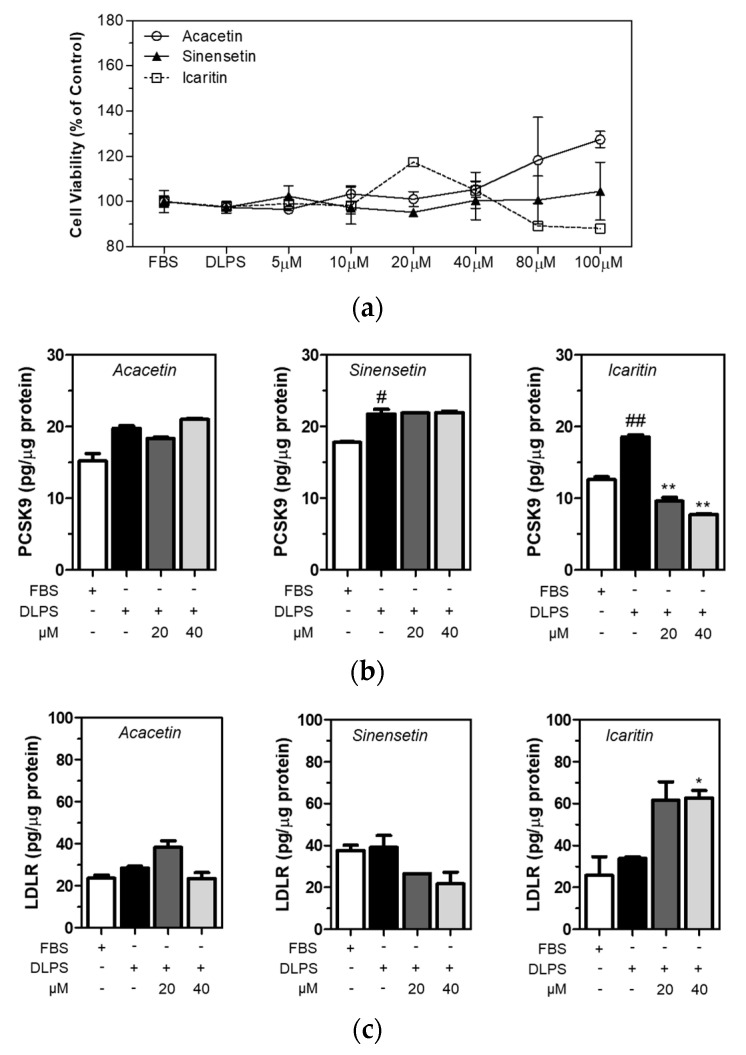
HepG2 cells were treated with FBS or DLPS in the presence or absence of acacetin, sinensetin, and icaritin (0, 5, 10, 20, 40, 80 µM), and MTT assay was performed to measure cell viability (**a**). The PCSK9 level in HepG2 cells treated with acacetin, sinensetin, and icaritin of up to 40 µM concentration was measured using ELISA (**b**). The LDLR level in HepG2 cells treated with acacetin, sinensetin, and icaritin of up to 40 µM concentration was measured using ELISA (**c**). significant difference between cells treated with FBS and DLPS (Student’s *t*-test, # *p* < 0.05; ## *p* < 0.01). significant difference between cells treated with DLPS and compounds (Student’s *t*-test, * *p* < 0.05; ** *p* < 0.01).

Acacetin, sinensetin, and icaritin are polyphenolic compounds found in capsella bursa-pastoris, although they have not been quantified [22]. In the present study, all three polyphenolic compounds in CBE were identified. It was found that sinensetin (106.6 ± 8.6 milligrams per kilogram of extract) was the most abundant, followed by acacetin (24.4 ± 1.2 mg/kg) and icaritin (17.5 ± 0.5 mg/kg) (Table 3). Chromatograms and standard curves are provided in Supplementary figures (Figures S1 and S2). Cell viability was measured using MTT assay, and the viability of the HepG2 cells treated with acacetin, sinensetin, and icaritin did not change significantly (Figure 3a). Intracellular PCSK9 and LDLR levels were measured using ELISA in HepG2 cells treated with acacetin, sinensetin, and icaritin of up to 40 µM concentration. DLPS increased PCSK9 level in HepG2 cells, and icaritin dose-dependently and significantly (*p <* 0.01) attenuated PCSK9 level in HepG2 cells under lipid depletion conditions (Figure 3b). In addition, a higher level of icaritin alone significantly increased the LDLR level in HepG2 cells treated with DLPS (Figure 3c). These findings together suggest that while sinensetin is the most abundant polyphenolic compound in CBE, icaritin has greater PCSK9 inhibitory activity compared to acacetin or sinensetin, and consequently, contributes to enhancing the LDLR level in HepG2 cells in lipid depletion conditions.

### 3.5. Icaritin Significantly Attenuated Gene Expression of PCSK9 and LDLR via SREBP2 and HNF-1α Downregulation

**Figure 4:**
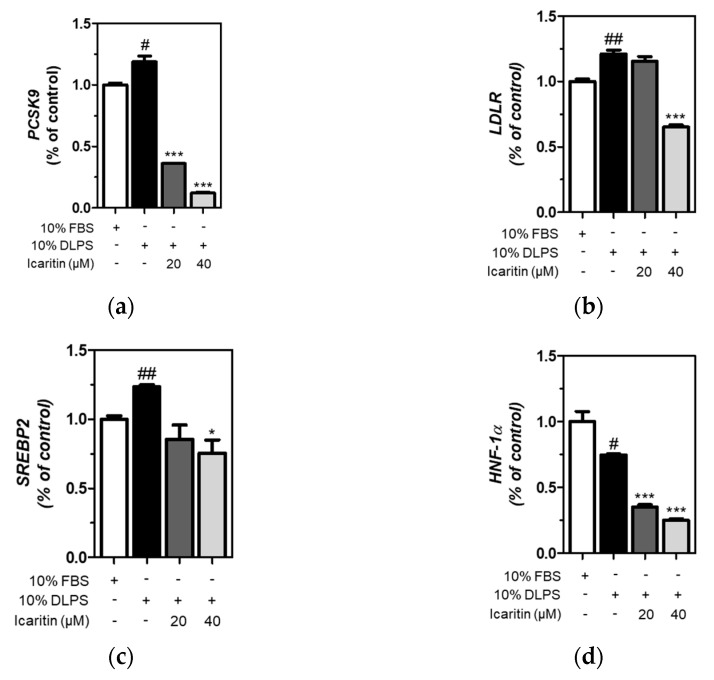
Expression of genes, including PCSK9 (**a**), LDLR (**b**), SREBP2 (**c**), and HNF-1α (**d**) in HepG2 cells treated with fetal bovine serum (FBS) or DLPS in the presence or absence of icaritin (0, 20, 40 µM), measured using qRT-PCR. significant difference between cells treated with FBS and DLPS (Student’s *t*-test, # *p* < 0.05; ## *p* < 0.01). significant difference between cells treated with DLPS and compounds (Student’s *t*-test, * *p* < 0.05; *** *p* < 0.001).

The expression of genes was measured using qRT-PCR, and we found that icaritin significantly (*p <* 0.001) attenuated DLPS-induced upregulation of *PCSK9* and *LDLR* gene expression (Figure 4a,b). DLPS significantly increased *SREBP2* gene expression, which was significantly (*p <* 0.05) attenuated by icaritin at 40 μΜ (Figure 4c). In addition, DLPS-mediated decreased *HNF-1α* gene expression was further significantly down-regulated by icaritin treatment (*p <* 0.001) in HepG2 cells (Figure 4d). Together, icaritin-mediated PCSK9 inhibition is attributed to the downregulation of *SREBP2,* as well as *HNF-1α* gene expression, in HepG2 cells treated with DLPS.

### 3.6. Icaritin Significantly Suppressed the Extracellular PCSK9 Level Increased by DLPS in HepG2 Cells

**Figure 5:**
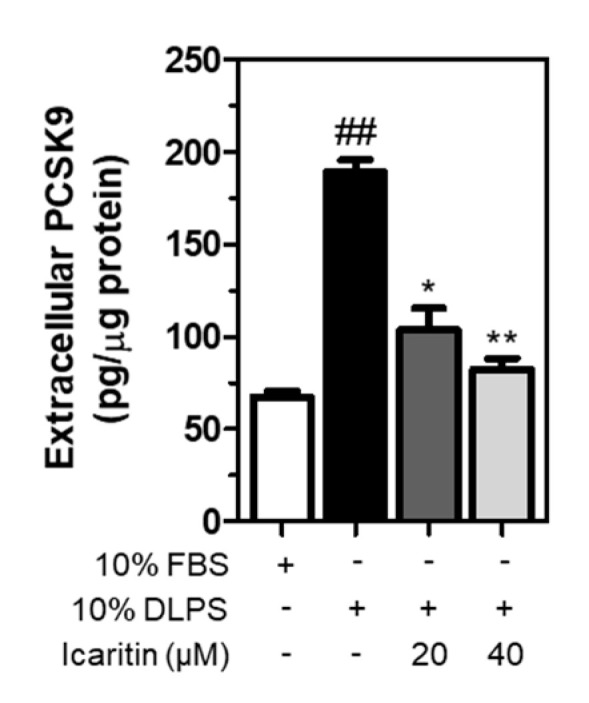
HepG2 cells were treated with FBS or DLPS in the presence of icaritin (0, 20, 40 µM), and extracellular PCSK9 was measured using ELISA. significant difference between cells treated with FBS and DLPS (Student’s *t*-test, ## *p* < 0.01). significant difference between cells treated with DLPS and compounds (Student’s *t*-test, * *p* < 0.05; ** *p* < 0.01).

Following secretion from the Golgi apparatus, a mature form of PCSK9 binds to LDLR, which is internalized and then transported with LDLR to the lysosomes for degradation [23]. In the present study, extracellular PCSK9 was measured in HepG2 cell culture media following treatment in lipid depletion conditions. As a result, DLPS significantly increased PCSK9 secretion in the media, which was decreased significantly and dose-dependently by icaritin (Figure 5). This suggests that icaritin reduces PCSK9 secretion, which in turn contributes to the prevention of LDLR lysosomal degradation.

## 4. Discussion

**Figure 6:**
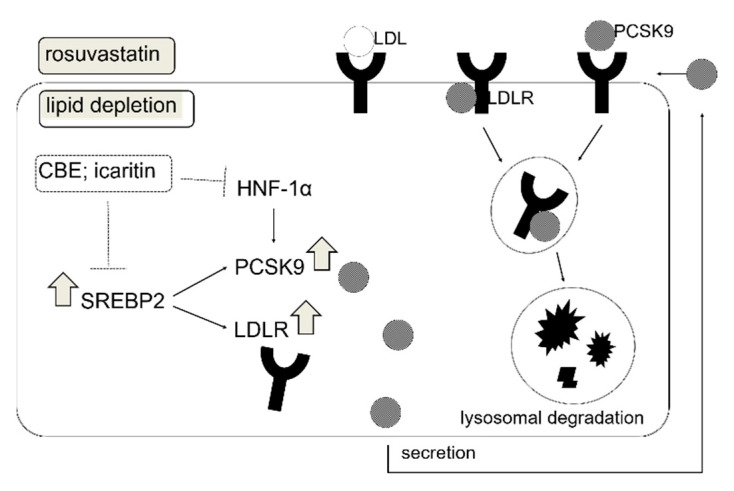
Schematic overview of cellular regulation of PCSK9 and LDLR by rosuvastatin and CBE and its active compound icaritin in lipid deficient conditions. Among the CBE components, icaritin increases LDLR by inhibiting PCSK9 via suppression of transcriptional activation of SREBP2 and HNF-1α.

The present study demonstrated that CBE had a cholesterol-lowering effect via PCSK9 inhibition. CBE-mediated cholesterol-lowering activity was observed in WD-fed obese mice via reduction in the hepatic *PCSK9* gene expression. Intracellular PCSK9 and LDLR protein levels were significantly increased in HepG2 cells when the cells were cultured in lipid-deficient media, which was significantly suppressed by the treatment of CBE (50 μg/mL). LDLR protein expression was unchanged by CBE, which can likely be attributed to PCSK9 downregulation, thereby preventing LDLR lysosomal degradation [24]. CBE-mediated PCSK9 inhibition was likely associated with decreased SREBP2 and HNF-1α in HepG2 cells. We also found that a combination of CBE and rosuvastatin increases LDLR protein expression, while suppressing the upregulation of PCSK9 induced by rosuvastatin. Three different phenolic compounds in CBE have been quantified using HPLC and evaluated to examine PCSK9 inhibitory activity. Among these compounds (acacetin, sinensetin, and icaritin), sinensetin is the most abundant in CBE, whereas icaritin (20 μM) showed significant PCSK9 inhibition. This indicates that icaritin was the most effective PCSK9 inhibitor. We further demonstrated that icaritin significantly reduced the effect of the PCSK9 and LDLR genes by inhibiting their transcription factors, *SREBP2* and *HNF-1α*, in HepG2 cells, together contributing to the suppression of PCSK9 secretion (Figure 6).

In the present study, HepG2 cells were grown in lipid-deficient media. Hunger or satiety could be an important factor for SREBP activation [25,26,27]. When the intracellular cholesterol level was depleted, a cholesterol sensor (SCAP) transports SREBP2 to Golgi apparatus, where proteolytic maturation of SREBP2 occurs. Then, mature SREBP2 enters the nucleus, where it leads to transcriptional activation of both PCSK9 and LDLR, since PCSK9 and LDLR both contain sterol regulatory element (SRE) motifs in their proximal promoters [28]. Another transcription factor contributing to the regulation of PCSK9 is HNF-1α. The HNF-1 binding site is located between the SRE and Sp1 site, and HNF-1α is predominant in the regulation of the PCSK9 gene in HepG2 cells [29]. Recently, it has been reported that statins stimulate HNF-1α expression, leading to increased production of PCSK9 [29,30].

Secreted extracellular PCSK9 post-translationally regulates the number of LDLRs on the cell surface. Following secretion, PCSK9 binds to the epidermal growth factor repeat A (EGF-A) region of the LDLR [31,32,33]. Icaritin significantly suppressed extracellular PCSK9 level in lipid-depleted media, supporting the role of icaritin in post-translational reduction of LDLR. Indeed, LDLR expression can be affected in many ways. While SREBP1α/2 are transcriptional regulators, the RING E3 ubiquitin ligase inducible degrader of the LDL receptor (IDOL, also known as MYLIP) acts as a post-transcriptional regulator of LDLR [34,35]. IDOL promotes ubiquitylation and subsequent lysosomal degradation of LDLR, thereby limiting the uptake of lipoprotein-derived cholesterol into the cells. A negative relationship between IDOL-LDLR plays an important role in circulating cholesterol homeostasis. Scotti et al. [36] generated homozygous mouse embryonic stem cells for a null mutation in the *IDOL* gene. Cells lacking *IDOL* showed marked increases in LDLR protein and LDL uptake rates. Scotti et al. [36] also found altered responses to multiple sterol metabolism regulators, such as serum oxysterols and synthetic liver X receptor (LXR) agonists in *IDOL*-null cells, independent of the SREBP pathway. Additionally, the deletion of *IDOL* (*IDOL*^(-/-)^; *IDOL*-KO) leads to reduced circulating levels of cholesterol, triglycerides, glucose, and insulin, and is responsible for decreased hepatosteatosis and fat mass in diet-induced obese mice [37]. Whether CBE and its bioactive components lower LDL cholesterol levels in relation to this LXR-IDOL-LDLR axis requires further investigation.

We have previously found that a Welsh onion ethanol extract and its active compound, kaempferol, acts as a PCSK9 inhibitor via SREBP2 downregulation in HepG2 cells. Since LDLR and PCSK9 both contain sterol regulatory elements within their promoter, SREBP2 can stimulate the transcription of both the LDLR and PCSK9 genes [24]. The transcription factor HNF-1α is another important transactivational mediator of PCSK9 that positively regulates PCSK9 gene expression. HNF-1α-binding element within the PCSK9 promoter has been reported to stimulate PCSK9 transcription in HepG2 cells [29]. Moreover, a number of studies have demonstrated that flavonoids can inhibit PCSK9 via HNF-1α downregulation, thereby exerting cholesterol-lowering activity. Curcumin [38], Pigeon Pea (Cajanus cajan (L.) Millsp.) [39], and berberine [40] have been reported as PCSK9 inhibitors via HNF-1α suppression.

We have previously demonstrated that CBE protects against high-fat diet (HFD)-induced hepatic steatosis by inhibition of HAT activity in HepG2 cells and HFD-induced obese mouse livers [3], suggesting that CBE acts as an HAT inhibitor. It has been demonstrated that LDLR can be epigenetically regulated through DNA hypo-methylation of CpG sites, and/or through the regulation of DNMT expression [41,42]. A study conducted by Benassi et al. [43] demonstrated that hazelnut treatment caused the hypomethylation of the LDLR promoter, which was concurrent with the observed increase in LDLR transcription and protein levels, suggesting that LDLR can be a direct target of bioactive components in food via a promoter DNA methylation-dependent mechanism. In the current study, CBE, acting as an HAT inhibitor, may mediate the epigenetic regulation of *LDLR* or other related genes, and thereby increase serum LDL cholesterol levels. This requires further study.

## 5. Conclusions

To the best of our knowledge, this is the first study to demonstrate the cholesterol-lowering activity of CBE as a PCSK9 inhibitor in diet-induced obese mice. CBE and icaritin inhibited PCSK9 via *SREBP2* and *HNF-1α* downregulation, thereby suppressing PCSK9 secretion in HepG2 cells. Increased circulating PCSK9 promotes hypercholesterolemia by binding LDLR, facilitating transport of LDLR to the lysosomes for degradation instead of recycling LDLR back to the cell surface [44]. Treatment of HepG2 cells with a combination of rosuvastatin and CBE further suppressed PCSK9 compared to cells treated with rosuvastatin alone, and increased LDLR protein expression compared to cells treated with CBE alone. Although icaritin is not the most abundant polyphenol found in CBE, its PCSK9 inhibitory activity was the most significant compared to others, including acacetin and sinensetin. Further study is required to demonstrate whether a physiological dosage of icaritin will exhibit hypocholesterolemic activity via PCSK9 inhibition and beyond mechanisms in a diet-induced obese mouse model. Our data suggest that CBE and icaritin might be interesting candidates for the treatment of hypercholesterolemic patients instead of statin therapy alone, for those not reaching their therapeutic goals.

## Figures and Tables

**Table 1:** Serum total and LDL cholesterol levels of the mice fed with LD, WD, or WD+1%CBE for 12 weeks.

	LD	WD	WD+1%CBE
Total Cholesterol (mg/dL)	94.85 ± 4.06	195.70 ± 7.24 ^###^	145.50 ± 3.23 ***
LDL Cholesterol (mg/dL)	12.69 ± 2.22	48.03 ± 6.77 ^###^	24.00 ± 3.54 **

^###^ indicates a significant difference between low-fat diet (LD) and western-type diet (WD) (Student’s *t*-test, *p* < 0.001). ** indicates a significant difference between WD and WD+1% ethanol extract of *Capsella bursa-pastoris* (CBE) (Student’s *t*-test, *p* < 0.01). *** indicates a significant difference between WD and WD+1%CBE (Student’s *t*-test, *p* < 0.001).

**Table 2:** Hepatic expression of genes, including LDLR, PCSK9, SREBP2, and HMGCR, in mice fed with LD, WD, or WD+1%CBE for 12 weeks.

	LD	WD	WD+1%CBE
*PCSK9*	2.45 ± 0.47	2.72 ± 0.57	0.94 ± 0.25 **
*LDLR*	1.37 ± 0.20	1.14 ± 0.20	1.35 ± 0.21
*SREBP2*	2.27 ± 0.40	2.13 ± 0.35	1.38 ± 0.24
*HMGCR*	2.02 ± 0.24	2.04 ± 0.27	1.19 ± 0.23 *

* indicates a significant difference between WD and WD+1%CBE (Student’s *t*-test, *p < 0.05*). ** indicates a significant difference between WD and WD+1%CBE (Student’s *t*-test, *p < 0.01*).

**Table 3:** Concentration of polyphenolic compounds in CBE (milligrams per kilogram of extract).

Polyphenolic Compounds	Concentration
Acacetin	24.4 ± 1.2
Sinensetin	106.6 ± 8.6
Icaritin	17.5 ± 0.5

## Data Availability

Not applicable.

## Supplementary Materials

The following are available online at https://www.mdpi.com/2304-8158/10/2/408/s1, Figure S1: High performance liquid chroma tograms of reference standards and CBE, Figure S2: Standard curves used for quantification of sinensetin, acacetin, and icaritin.
