# Supplementary material for: The Cholesterol-Lowering Effect of Capsella Bursa-Pastoris Is Mediated via SREBP2 and HNF-1α-Regulated PCSK9 Inhibition in Obese Mice and HepG2 Cells
Source: Foods. 2021 Feb 12;10(2):408. doi: 10.3390/foods10020408 (PMC7918551; doi:10.3390/foods10020408)
Supplement: Supplementary file 1 [file foods-10-00408-s001.pdf]

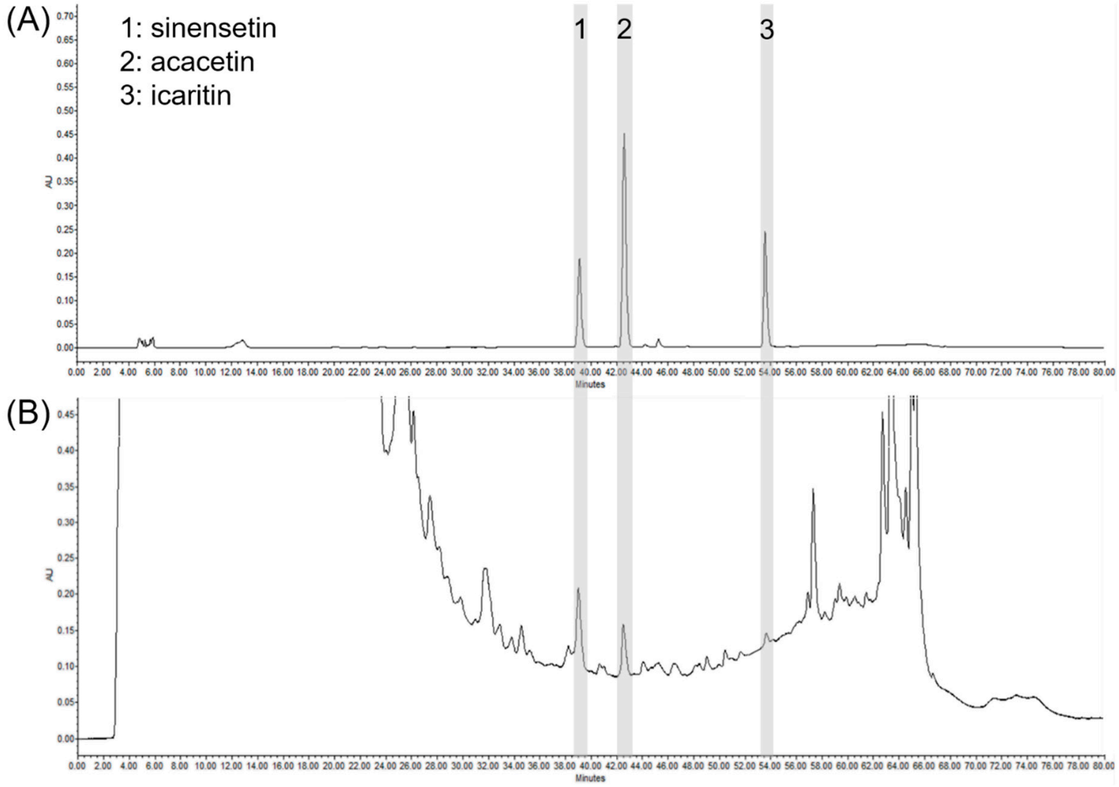

Figure 1. High performance liquid chromatography chromatograms of reference standards (A) and *Capsella bursa-pastoris* ethanol extract (B).

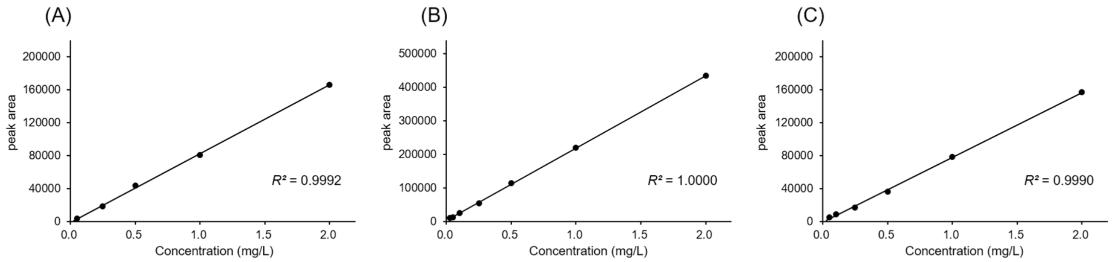

Figure 2. Standard curves used for quantitation of sinensetin (A), acacetin (B), and icaritin (C).
